# An extensive posterior circulation infarction secondary to primary hyperthyroidism accompanied with superior mesenteric artery syndrome: A case report and description of patho-physiological association

**DOI:** 10.1097/MD.0000000000022664

**Published:** 2020-11-13

**Authors:** Hong-Kai Wang, Wen-Hsuan Huang, Ko-Ting Chen

**Affiliations:** a Department of Neurosurgery, Chang Gung Memorial Hospital at Linkou; b Department of General Surgery, Saint Paul's Hospital; c Ph.D. Program in Biomedical Engineering, Chang Gung University, Taoyuan, Taiwan.

**Keywords:** cerebellar infarction, Graves disease, hyperthyroidism, posterior circulation infarction, superior mesenteric artery syndrome

## Abstract

**Introduction:**

Hyperthyroidism-related anterior circulation ischemic events have been well documented; however, posterior circulation infarction is rarely reported, not to mention with superior mesenteric artery syndrome (SMAS), which has never been reported concurrently. We describe, to the best of our knowledge, the first case of hyperthyroidism-related cerebellar infarction accompanied with SMAS.

**Patient concerns:**

A 22-year-old women presented with palpitation, postprandial vomiting, and acute body weight loss. Enlarged thyroid gland was discovered in physical examination and Graves disease was diagnosed by blood test; therefore, Propylthiouracil and β-blocker were prescribed. Sudden onset conscious disturbance accompanied with apnea was noted during hospitalization.

**Diagnosis:**

Computed tomography (CT) revealed cerebellar infarction with severe cerebellar swelling and tonsil herniation; hence, emergent suboccipital craniotomy and bilateral tonsillectomy were performed.

**Interventions:**

Nevertheless, persisted poor passage of liquid diet during nasogastric tube feeding was noted after operation. CT of abdomen showed a sharp aorta-SMA angle (15°) and a short distance between aorta and SMA (6 mm) indicating a diagnosis of SMAS.

**Outcomes:**

After parental nutrition supplement and progressive rehabilitation program, she recovered to a modified Rankin Scale of 3.

**Conclusion:**

Although rarely reported, hyperthyroidism-related sympathetic hyperstimulation, vasculopathy could result in potentially deadly posterior circulation infarction. Furthermore, SMAS should be considered in the cases of hyperthyroidism with prolonged gastrointestinal symptoms even after treatment and should be treated simultaneously, since SMAS exacerbates depletion of intravascular volume. Further study to clarify the relation between hyperthyroidism and posterior circulation hemodynamic status is suggested.

## 1 Introduction

Hyperthyroidism may be asymptomatic or it can present with weight loss, anxiety, increased heart rate, hyperactive bowel movement, heat intolerance, tremor, fatigue, and so on.^[1]^ The most common cause of hyperthyroidism is Graves disease (GD) which is an autoimmune disorder in which autoantibodies to the thyroid stimulating hormone (TSH) receptor induce continuous stimulation of thyroid gland.^[2]^ Hyperthyroidism could be associated with stroke through mechanisms including atrial fibrillation-related embolism, moyamoya syndrome (MMS), vasculopathy, and vasospasm.^[2–5]^ Moyamoya syndrome is characterized by progressive stenosis and occlusion of bilateral or unilateral terminal portions of internal carotid artery with associated causative systemic conditions.^[6–8]^ Despite rarely the cause of MMS, the association between GD and MMS has been well documented.^[2,7–10]^ Several hypotheses have been postulated for hyperthyroidism-related vasculopathy including sympathetic nervous system stimulated by thyroid hormone which may cause atherosclerotic changes in vessel, and autoimmune antibodies that may cause vascular local inflammation.^[2,6]^ A recent study by Chen et al has shown the incidence of disease progression in patients with MMS with GD was significantly higher than that in patients with moyamoya disease without GD, indicating a contributory role of GD in ischemic stroke and an association between GD and MMS.^[2]^ Although anterior circulation vasculopathy concurrent with GD has been well reported, posterior circulation disease concurrent with GD is rare to be mentioned.^[2,3,6,8]^

Superior mesenteric artery syndrome (SMAS) causes proximal intestinal obstruction due to third portion or the final portion of duodenum trapped between aorta and SMA.^[11,12]^ The most common presentations are abdominal pain, nausea, vomiting, anorexia, and weight loss.^[13]^ Konen et al^[12]^ have measured a normal range of aorta-SMA angle and aorta-SMA distance using computed tomographic angiography (CTA) and a decrease of both values were reported in SMAS due to decrease of mesenteric fat. Only 1 case reported the association between GD and SMAS in the literature, in which GD serve as an aggravating factor to the decrease of mesenteric fat.^[14]^

Herein, we report a patient with newly diagnosed GD suffered from acute extensive bilateral cerebellar infarction who underwent emergent suboccipital craniectomy and SMAS was diagnosed later on. We also describe the patho-physiological association between GD, SMAS, and posterior circulation infarction in our case.

## 2 Case Presentation

A 22-year-old woman presented with palpitation, anorexia, and postprandial vomiting for 1 month. She lost 8 kg of body weight in 3 months. Physical examinations revealed bilateral enlarged thyroid gland. Her blood tests were consistent with primary hyperthyroidism (T3: 318.59 ng/dL, normal range: 58–159 ng/dL; free T4: 3.84 ng/dL, normal range: 0.7–1.48 ng/dL; TSH: <0.012 μIU/mL, normal range: 0.35–4.94 μIU/mL). The anti-THS receptor antibody level was 6.6 IU/L (normal range <1.75 IU/L), indicating a Graves disease. Under the impression of acute thyrotoxicosis, medications including Propylthiouracil (PTU) and β-blocker were prescribed; however, she became so weak rapidly in days that she was hospitalized for a survey of refractory postprandial vomiting.

**Figure 1:**
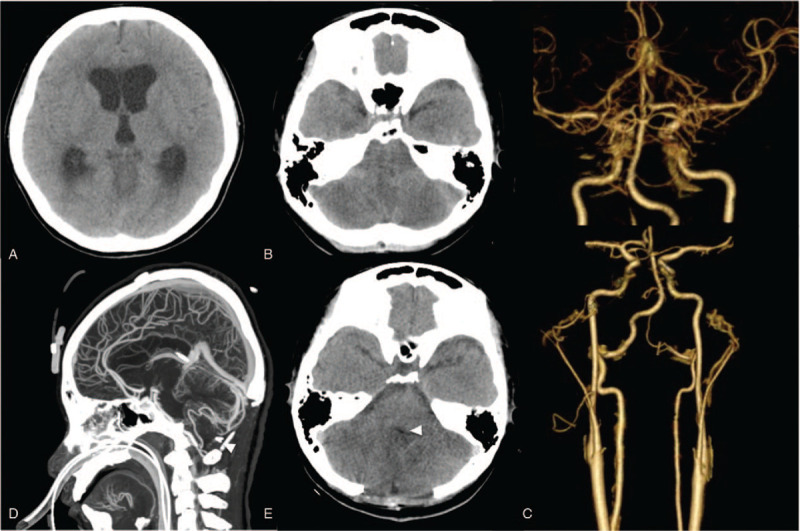
Pre- and postoperative brain CT/CT angiography. A preoperative brain CT showed severe obstructive hydrocephalus with ventriculomegaly (A) and low signal density of bilateral cerebellum with tight posterior fossa (B), indicating acute cerebellar infarction with brain stem compression. After emergent suboccipital craniotomy, a postoperative brain CT angiography was performed to investigate vascular patency (C–E). The anterior circulation was intact with vascular compromised; however, the left intracranial vertebral artery (VA) hypoplasia was noted and bilateral superior cerebellar arteries (SCA), anterior inferior cerebellar arteries (AICA), and posterior inferior cerebellar arteries (PICA) were vanished, suggesting a bilateral SCA, AICA, and PICA territories infarction. After craniectomy, the posterior fossa was decompressed till foramen magnum (D, arrowhead) and the fourth ventricle was patent (E, arrowhead). CT = computed tomography.

A rapid onset of dyspnea, then apnea and conscious disturbance with flaccid paralysis of 4 limbs was found the day after admission. Her blood pressure was 65/40 mm Hg and Glasgow coma scale was E1V1M1. After emergent intubation, a brain computed tomography (CT) revealed bilateral cerebellar hypodensity lesions with tonsil herniation, combined with acute obstructive hydrocephalus and severe brain swelling (Fig. 1, A and B). An emergent suboccipital craniectomy, bilateral tonsillectomy with dura augmentation for brain stem decompression, and an external ventricular drainage tube insertion for cerebrospinal fluid diversion were performed. At intensive care unit, a brain CTA revealed poor contrast filling of bilateral superior cerebellar arteries (SCA), anterior inferior cerebellar arteries (AICA), and posterior inferior cerebellar arteries (PICA), and asymmetric vertebral arteries (VA) (Fig. 1, C–E). A young stroke survey including cardiac echocardiography that was negative of embolism and a survey for possible causes of vasculitis in posterior circulation and cerebellitis revealed negative finding of acute infections, including mumps, varicella zoster virus, measles, Epstein-Barr virus, venereal disease research laboratory, herpes simplex virus, Coxsackie virus B, Chlamydophila, Mycoplasma, tumor markers, autoimmune screenings: antiphospholipid antibodies, antineutrophil cytoplasmic antibody (ANCA), paraneoplastic antibodies, anti-aquaporin-4 antibody, limbic encephalitis antibodies, antiglutamic acid decarboxylase antibody, antityrosine phosphatase-like insulinoma antigen 2 antibody. Hyperthyroidism-related vasculopathy was suspected to be the culprit for such diffuse cerebellar infarction.

**Figure 2:**
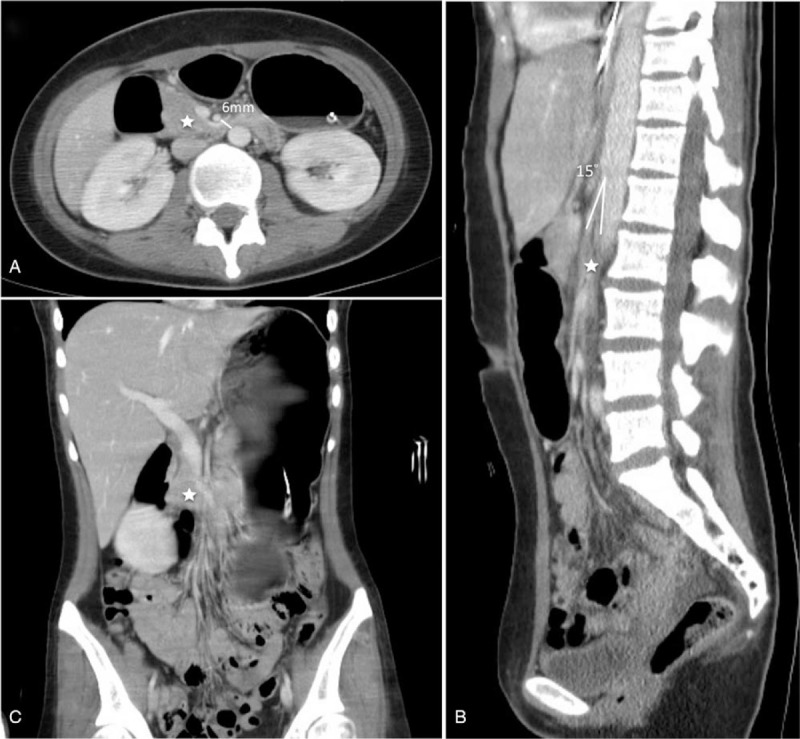
A brain MRI showed bilateral extensive signal hyperintensity in T2-weighted imaging, indicating a previous insult of cerebellar hemispheric infarction at SCA and PICA territories bilaterally. PICA = posterior inferior cerebellar artery, SCA = superior cerebellar artery, MRI = magnetic resonance imaging.

Her consciousness gradually improved to E3VeM6 in 2 weeks postoperatively. However, daily feeding from nasogastric tube could not achieve appropriate caloric goal due to milk stasis in the stomach. Upon percussion and auscultation, tympanic sound and intermittently hypermobile gastric movement were found. Abdominal CT showed a bloated stomach and the horizontal portion of the duodenum was sandwiched between the aorta and the SMA, causing duodenal obstruction (Fig. 2, B and C). The aorta-SMA angle was approximately 15°, while the aorta-SMA distance was 6 mm (Fig. 2, A and B). Repeated endoscopic naso-jejunal tube insertion was failed to bypass the SMA impinged duodenum. Parental nutrition was given for prolonged poor enteral feeding-related significant body weight loss. She underwent tracheostomy and was transferred to respiratory care unit for weaning ventilator.

**Figure 3:**
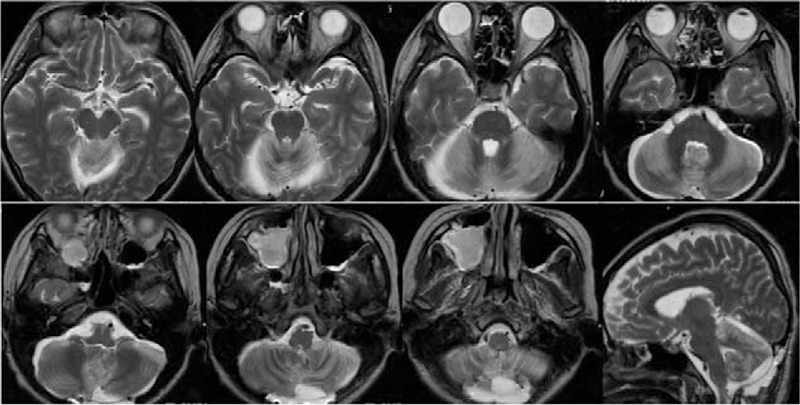
A contrast-enhanced abdominal CT suggested superior mesentery artery (SMA) syndrome. The axial view (A) demonstrated a distance between SMA and abdominal aorta was 6 mm and the proximal part duodenum third portion (asterisk) was dilated while the distal part was compressed. In sagittal view (B), the aorta-SMA angle was 15°. In coronal view, a bloated stomach occupied left upper abdominal quadrant and an obstructed duodenum can be seen (asterisk). CT = computed tomography.

A slow but progressive rehabilitation program was arranged for her after a gradual improvement of enteral feeding and gain of body weight 3 months postoperatively. One and half year after the operation, she had symmetric muscle power of at least grade 4/5 and was able to walk with minimal assistance with a sequela of mild ataxic gait. Her tracheostomy was closed and she was able to talk with a scanning speech. A follow-up magnetic resonance imaging of brain showed reveal an insult of extensive cerebellar hemispheric injury with a relative preservation of deep nuclei of cerebellum (Fig. 3). Finally, she recovered to a modified Rankin Scale of 3.

## 3 Discussion

To the best of our knowledge, this is the first reported case of combined GD and SMAS presenting as acute extensive cerebellar infarction in the literature, and with a properly and promptly therapeutic and diagnostic strategies, a satisfactory outcome was achieved. We discuss the relations between GD and cerebellar infarction and GD and SMAS, and finally propose a description of the patho-physiological association among these factors to the result of cerebellar infarction.

### 3.1 GD and cerebellar infarction

Graves disease has been an established cause of MMS, indicating ischemia or infarction of anterior circulation,^[2,3,6,8–10]^ however, it is extremely rare, in fact, only 1 case was reported in the literature, to find GD concurrent with posterior circulation infarction.^[15]^ In our case, a postoperative magnetic resonance imaging confirmed the infarcted area including bilateral SCA and PICA territories. Since multiple vascular lesions occurred simultaneously, vasculitis and hyperthyroidism-related vasculopathy were suspected in priority. PTU-induced ANCA-related vasculitis has been reported,^[4,16]^ however, the blood test showed negative of eosinophilia, perinuclear-ANCA, or antinuclear antibody. As there was no atrial fibrillation noted during hospitalization and examination, atrial fibrillation-related embolism was not likely. Autoimmune assay for cerebellitis and coagulopathy survey for hypercoagulable status were also performed but results were all negative. VA hypoplasia has been reported to be associated with ipsilateral PICA hypoperfusion,^[17]^ however, this could not explain the bilateral symmetric involvement in our patient. Hyperthyroidism may result in sympathetic hyperstimulation and sympathetic hypersensitivity which lead to various vasculopathy and possible vasospasm.^[2,3,6,9]^ Sympathetic hyperstimulation may deteriorate posterior circulation hypoperfusion in the cases with posterior circulation vascular anomaly.

### 3.2 GD and SMAS

The main cause of SMAS is considered to be acute loss of body weight and a reduction of body fat.^[14]^ A direct association of GD and SMAS has not been reported. Hirai et al have reported a patient with SMAS complicated by diabetic ketoacidosis and GD, in which they preferred diabetic ketoacidosis as the main contributor to acute body weight loss while similar cases have been reported.^[14,18–20]^ In our patient, an untreated GD was the only systemic disease causing her SMAS, since she had nausea and weight loss before her presentation to the clinic. Therefore, this might be the first case to demonstrate an association between GD and SMAS to the best of our knowledge. In addition, presentations of hyperthyroidism might be similar to SMAS including weight loss, diarrhea, nausea, and vomiting.^[11,13]^ In our case, 8 kg of body weight loss was noted in 3 months and her gastrointestinal symptoms persist even after anti-hyperthyroidism medications. The exact occurrence of SMAS could not be assured; however, an obstructed gastropathy-related poor oral intake would exacerbate SMAS.^[11]^ Therefore, in cases of hyperthyroidism with sudden body weight loss accompanied with gastrointestinal symptoms, SMAS should be taken into consideration and should be treated simultaneously.

### 3.3 A patho-physiological association between GD, SMAS, and cerebellar infarction

**Figure 4:**
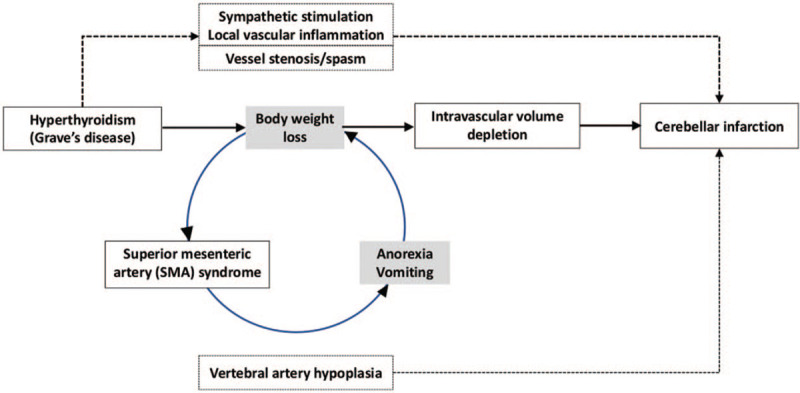
A flow diagram to delineate the possible pathophysiology between hyperthyroidism, SMAS, and cerebellar infarction. Hyperthyroidism-related vasculopathy, vascular wall inflammation, and sympathetic stimulation have been proposed to be directly related to cerebellar infarction. SMA syndrome was related to visceral fat decrease and impingement of duodenum by aorta-SMA angle, which further worsened the already decreased body weight and visceral body fat, further depleted intravascular volume, and aggravated cerebellar ischemia/infarction. A hypoplasia of left vertebral artery may attribute to cerebellar infarction despite a minor role. SMAS = superior mesenteric artery syndrome.

We propose a flow diagram to delineate the association between GD, SMAS, and cerebellar infarction (Fig. 4). GD-related body weight loss may cause SMAS, which exacerbate anorexia and vomiting, further decrease body weight. The vicious cycle could result in dramatic depletion of intravascular volume which alters cerebrovascular hemodynamic condition and causes intracranial hypoperfusion. Besides, hyperthyroidism-related hypersensitivity to sympathetic signal may lead to local vascular inflammation and vessel stenosis; while VA hypoplasia-related asymmetric flow may also aggravate perfusion decrement. Eventually, a devastating cerebellar infarction occurred.

While hyperthyroidism-associated anterior circulation infarction has been well documented, very few research describe the relationship between hyperthyroidism and posterior circulation infarction, not to mention the situation accompanied with SMAS. We reported a successful treatment of an extremely rare case of extensive cerebellar infarction accompanied with GD and SMAS. Further studies are required to evaluate hemodynamic change in patients with hyperthyroidism and posterior circulation.

## Author contributions

**Conceptualization:** Wen-Hsuan Huang, Ko-Ting Chen.

**Data curation:** Hong-Kai Wang, Wen-Hsuan Huang, Ko-Ting Chen.

**Methodology:** Hong-Kai Wang.

**Supervision:** Ko-Ting Chen.

**Writing – original draft:** Hong-Kai Wang, Ko-Ting Chen.

**Writing – review & editing:** Hong-Kai Wang, Wen-Hsuan Huang, Ko-Ting Chen.
